# Advanced Metabolic-Guided Perfusion Concepts in an Obese Patient Undergoing Cardiac Surgery: A Case Report

**DOI:** 10.7759/cureus.99447

**Published:** 2025-12-17

**Authors:** Ali Civelek, Alireza Soltanzadeh, Maryam Alizadegan

**Affiliations:** 1 Department of Cardiovascular Surgery, Okan University Hospital, Istanbul, TUR; 2 Department of Anesthesiolgy, Okan University Hospital, Istanbul, TUR

**Keywords:** cardiopulmonary bypass, do₂/vco₂ ratio, goal-directed perfusion, obesity, oxygen delivery (do₂)

## Abstract

Obesity increases metabolic oxygen demand, impairs microvascular reserve, and reduces tolerance to hemodilution during cardiopulmonary bypass (CPB), making adequate indexed oxygen delivery (DO₂i) especially important. Evidence on DO₂i-guided perfusion in obese patients undergoing redo cardiac surgery remains limited. We report the case of a 61-year-old obese woman (125 kg; BSA 2.23 m²) undergoing redo mitral valve replacement and tricuspid repair. Her elevated oxygen requirements and prolonged CPB time placed her at high risk for oxygen-delivery instability. Continuous metabolic monitoring integrated DO₂i, indexed carbon-dioxide production (VCO₂i), oxygen consumption index (VO₂i), mixed venous oxygen saturation (SvO₂), temperature-corrected oxygen-extraction ratio (O₂Eri), and the DO₂/VCO₂ ratio. Because the procedure was performed at 30°C, DO₂i was adjusted using a Q₁₀-based correction to reflect reduced metabolic demand under moderate hypothermia. A goal-directed perfusion strategy targeting a temperature-corrected DO₂i ≥ 300 mL/min/m² enabled early detection and correction of transient DO₂i declines (nadir 260 mL/min/m²). Cumulative AUC-DO₂ remained <1,000 mL/min/m²/min, with minimal exposure to DO₂i <280 mL/min/m². Aerobic metabolism was preserved throughout CPB, postoperative lactate stayed low, no acute kidney injury developed, and recovery was uncomplicated. This case shows that integrating DO₂i with advanced metabolic indices and temperature-adjusted analysis can enhance predictive perfusion management in obese high-risk patients. Further research is needed to define optimal DO₂i thresholds and temperature-specific GDP strategies for this population.

## Introduction

Obesity is an expanding global health burden and a well-recognized risk factor for adverse outcomes in cardiac surgery. Patients with elevated body mass index present a constellation of physiologic challenges, including increased metabolic oxygen consumption (VO₂i), reduced microvascular reserve, impaired pulmonary mechanics, and heightened susceptibility to hemodilution that narrows the margin of safety for maintaining adequate oxygen delivery during cardiopulmonary bypass (CPB) [1]. These intrinsic characteristics make oxygen metabolic management a central determinant of perioperative stability in obese individuals. Indexed oxygen delivery (DO₂i), which reflects the combined contribution of pump flow, hemoglobin concentration, and arterial oxygen content, is the principal perfusion variable used to ensure sufficient tissue oxygen supply [2]. Several clinical and experimental studies have established that DO₂i values below approximately 260-300 mL/min/m² are associated with increased risk of anaerobic metabolism, lactate accumulation, oxygen debt, and acute kidney injury [3,4]. Additional metabolic markers, including mixed venous oxygen saturation (SvO₂), oxygen-extraction ratio (O₂ERi), indexed carbon-dioxide production (VCO₂i), and the DO₂/VCO₂ ratio, further refine the assessment of metabolic adequacy by quantifying the balance between oxygen delivery and demand [5]. Collectively, these indices provide a more comprehensive representation of global aerobic stability than DO₂i alone. Despite these advances, the application of structured DO₂i-guided perfusion strategies in obese cardiac surgery patients remains poorly documented. Most available evidence derives from mixed or predominantly non-obese cohorts, which may not reflect the unique metabolic requirements of patients with severe obesity [1]. Importantly, these individuals may require higher minimum DO₂i thresholds, potentially exceeding 300 mL/min/m², and stricter limits on oxygen extraction and cumulative oxygen-delivery deficit, given their elevated VO₂i and limited physiologic reserve [6]. The absence of dedicated investigations limits the development of evidence-based perfusion protocols specifically tailored to this high-risk population, which is more prone to rapid deterioration during low-oxygen delivery states [7]. Traditional perfusion approaches, typically based on fixed flow indices, intermittent laboratory measurements, and delayed recognition of metabolic deterioration, may not identify early declines in DO₂i. Obese patients can transition more quickly into anaerobic metabolism because of their increased metabolic demands and reduced compensatory capacity, underscoring the need for continuous and anticipatory monitoring rather than reactive intervention [8]. A structured DO₂-centered strategy may therefore offer a more reliable means of preventing oxygen-debt accumulation and reducing end-organ vulnerability. Given the rising prevalence of obesity and the limited availability of targeted data, there is a clear need for clinical reports describing the practical implementation of DO₂-focused strategies in obese cardiac surgery patients. This case report presents the use of a multiparametric, DO₂i-guided perfusion approach in a severely obese patient undergoing complex redo cardiac surgery. By highlighting how continuous metabolic monitoring supported the preservation of aerobic metabolism and minimized cumulative oxygen debt, this report aims to contribute to the evolving understanding of oxygen-delivery management in a physiologically vulnerable population.

## Case presentation

A 61-year-old obese woman weighing 125 kg (BMI 45.9 kg/m²; BSA 2.23 m²) with a history of type 2 diabetes mellitus, hypertension, dyslipidemia, and mild obstructive sleep apnea was scheduled for redo cardiac surgery. She had previously undergone atrial septal defect (ASD) repair, resulting in dense mediastinal adhesions and altered venous anatomy. Baseline renal function was normal, with a creatinine concentration of 0.68 mg/dL and an estimated GFR. Her metabolic profile and body habitus placed her at high risk for oxygen-delivery instability and hemodilution-related complications during CPB.

Surgical technique

Redo cardiac surgery was performed through a standard median sternotomy. Dense retrosternal adhesions from the previous ASD repair required meticulous and time-consuming adhesiolysis to safely expose the cardiac structures. After completing the dissection, CPB was initiated using standard aorto-bicaval cannulation. Arterial access was obtained with a 22 Fr EOPA (Medtronic, Minneapolis, MN) aortic cannula placed in the ascending aorta, while venous drainage was achieved using bicaval cannulation (32 Fr and 28 Fr cannulae), allowing for stable flows and effective decompression. A ventricular vent cannula (13 Fr) was inserted through the right superior pulmonary vein, together with a pericardial sump for blood evacuation. An autotransfusion system was used throughout the procedure. Myocardial protection was achieved with antegrade Del Nido cardioplegia delivered through the aortic root, providing a prolonged period of diastolic arrest and stable electrical silence during valve exposure. The surgical repair involved a mitral valve replacement using a 31-mm Mosaic Bioprosthesis (Medtronic, Minneapolis, MN) tissue prosthesis and tricuspid annuloplasty with a 32-mm Contour 3D Annuloplasty Ring (Medtronic, Minneapolis, MN), ensuring durable reconstruction of both atrioventricular valves. Hemostasis was carefully optimized before weaning from CPB.

Anesthesia management

During CPB, volatile-based anesthesia was continued using sevoflurane delivered through the oxygenator vaporizer, ensuring adequate hypnotic depth throughout extracorporeal circulation. The minimum alveolar concentration (MAC) was maintained between 0.80 and 1.0, providing stable anesthetic coverage while supporting hemodynamic and metabolic stability.

Anticoagulation management

Systemic anticoagulation for CPB was achieved using unfractionated heparin at an initial dose of 300 IU/kg, titrated to maintain an activated clotting time (ACT) of 450 in accordance with institutional standards. ACT measurements were obtained at regular intervals throughout CPB to ensure stable anticoagulation. At the completion of bypass, anticoagulation was reversed with protamine sulfate administered at 600 U/kg, resulting in normalization of ACT without hemodynamic instability or coagulation-related complications.

Perfusion technique

CPB was performed using the Quantum Perfusion System (Spectrum Medical Ltd), which provided continuous real-time monitoring of DO₂i, VO₂i, carbon-dioxide production (VCO₂i), mixed venous oxygen saturation (SvO₂), and O₂ERi. The extracorporeal circuit consisted of a modular miniaturized adult configuration, incorporating a Terumo FX25 oxygenator and a 1,150 mL priming volume, reducing hemodilution and improving metabolic stability. Systemic temperature was maintained at 30 °C, necessitating adjustment of oxygen-delivery calculations for hypothermia. Using the Formula Editor integrated within the Quantum platform, a dedicated algorithm was applied to compute temperature-corrected DO₂i (formula presented by El Dsouki and Condello) [9], displayed concurrently with the standard DO₂i referenced to 37 °C. This dual-parameter visualization allowed optimization of perfusion based on both actual metabolic demand and the standardized physiologic reference, preventing misinterpretation of perfusion adequacy during hypothermia.

Temperature-corrected DO₂i (formula presented by El Dsouki and Condello) is as follows:

$\mathrm{taDO}_{2i} = f(T) \cdot \mathrm{BSA}$

f(T) is a quadratic function empirically derived through regression fitting from published physiological data and simulations. The regression coefficients were identified using curve-fitting on a synthetic dataset representing critical DO₂i at various temperatures (24-37°C), informed by both exponential and linear modeling techniques. The selected coefficients provided the best fit with minimal residual error, ensuring both biological plausibility and computational efficiency [8]. The perfusion management adhered to a fully DO₂-guided strategy, consistent with the “aerobic mix” framework. The primary intraoperative metabolic targets included (Table 1).

**Table 1:** Metabolic and Hemodynamic Targets During Cardiopulmonary Bypass

Parameter	Target Value	Units	Description
Indexed Oxygen Delivery (DO₂i)	> 280 (optimal 300)	mL/min/m²	Minimum DO₂i required to prevent anaerobic metabolism.
DO₂i / VCO₂i Ratio	> 5.3	-	Indicator of adequate aerobic reserve.
Indexed Oxygen Extraction Ratio (O₂ERi = VO₂i/DO₂i)	< 39%	%	Reflects balance between metabolic demand and oxygen delivery.
Indexed Carbon Dioxide Production (VCO₂i)	< 60	mL/min/m²	Threshold associated with stable aerobic metabolism.
Cumulative Oxygen Delivery Deficit (AUC–DO₂)	< 1,000	mL/min/m²/min	Preferred maximum cumulative exposure to low-DO₂ states.
Maximum Exposure to DO₂i < 280 mL/min/m²	< 15	min	Time-dependent tolerance limit for hypoxic delivery.
Mixed Venous Oxygen Saturation (SvO₂)	> 75%	%	Marker of global oxygen reserve and adequate perfusion.
Mean Arterial Pressure (MAP)	75-90	mmHg	Optimal pressure range to maintain organ perfusion.
Hemoglobin (Hb)	> 8.5	g/dL	Ensures adequate oxygen-carrying capacity.
Pump Flow Adjustment	Proportional to venous return	L/min	Flow titration to maintain stable DO₂i and optimize drainage.

Targets during CPB

Initial DO₂i values ranged between 285-295 mL/min/m², with lactate at 1.1 mmol/L, indicating an aerobic metabolic profile. Temperature-corrected DO₂i values were slightly lower, as expected under moderate hypothermia, allowing for more accurate interpretation of oxygen sufficiency relative to the reduced metabolic background at 30 °C. Continuous visualization of DO₂i (corrected and uncorrected), DO₂/VCO₂ ratio, VO₂i, VCO₂i, O₂ERi, and SvO₂ via the Quantum metabolic dashboard enabled rapid detection of oxygen-delivery decline. Interventions were implemented before significant oxygen-debt accumulation, as confirmed by a cumulative AUC-DO₂ < 1,000 mL/min/m²/min and exposure to critical DO₂i values (<280 mL/min/m²) for only 8.5 minutes, well below the 15-minute threshold considered physiologically tolerable. This integrated metabolic approach enhanced the predictive capability of GDP, reduced perfusion-related stress, and contributed to the patient’s stable postoperative outcome.

Events during CPB

During the initial phase of CPB, pump flow was set to 2.4 L/min/m², resulting in a starting DO₂i of approximately 285-295 mL/min/m². Hemoglobin at initiation measured 8.2 g/dL, and lactate remained low at 1.1 mmol/L, indicating preserved aerobic metabolism. Approximately 20 minutes into bypass, the perfusion team observed the first significant decline in oxygen delivery, with DO₂i falling to 260 mL/min/m². The drop was attributed to reduced venous return, most likely related to the patient’s body habitus and previous sternotomy. Corrective maneuvers were immediately implemented: pump flow was increased to 2.6 L/min/m², cannula positioning was optimized, and venous return was improved by placing the patient in a slight Trendelenburg position. These interventions rapidly restored DO₂i to the target range of 290-300 mL/min/m². A second DO₂i decline occurred later during bypass, this time driven by hemodilution. Hemoglobin decreased to 7.9 g/dL, causing DO₂i to fall to approximately 275 mL/min/m². Based on real-time oxygen-delivery data, the perfusionist increased the blood flow, and DO₂i rose promptly to 310-320 mL/min/m², fully re-establishing metabolic stability. Overall, DO₂i remained within the predefined target for 93% of the total CPB duration. The lowest recorded value was 260 mL/min/m², and the total time spent below 300 mL/min/m² was less than five minutes. Lactate peaked at only 1.8 mmol/L, confirming the absence of significant anaerobic metabolism throughout the procedure. The cumulative oxygen-delivery deficit (AUC-DO₂) remained below 1,000 mL/min/m²/min, indicating minimal oxygen debt. For the remainder of CPB, metabolic parameters remained stable. Mean DO₂i was 295 ± 10 mL/min/m², with SvO₂ sustained at 76 ± 2%, VO₂i at 68 ± 5 mL/min/m², VCO₂i at 58 ± 5 mL/min/m², and an oxygen-extraction ratio of 0.23 ± 0.02. After correction for procedural CO₂ administration, the DO₂/VCO₂ perfusion ratio remained in the aerobic range at 5.5 ± 0.38. Overall, the patient maintained fully aerobic metabolism for more than 95% of the bypass duration (Table 1). CPB and cross-clamp times were 156 and 113 minutes, respectively (Table 2).

**Table 2:** Goal-Directed Perfusion (GDP) and Intra-operative Data

Variables	Mean Value (± SD)
Pump Flow	5.4 ± 0.25 L/min
MAP	86 ± 4 mmHg
Hematocrit	26.3 ± 0.3%
PaO₂	230 ± 25 mmHg
PaCO₂	38 ± 2 mmHg
SaO₂	98 ± 0.5%
SvO₂	76 ± 2%
DO₂i	295 ± 10 mL/min/m²
VO₂i	68 ± 5 mL/min/m²
VCO₂i	58 ± 5 mL/min/m²
O₂ Extraction Ratio	0.23 ± 0.02
DO₂/VCO₂ Ratio	5.5 (corrected for CO₂ administration)
DO₂i < 280 mL/min/m²	8.5 min
AUC–DO₂	< 1,000 mL/min/m²/min
Bypass Time	156 min
Aortic Cross-Clamp Time	113 min

Postoperative course

Postoperatively, the patient remained hemodynamically stable without requiring vasoconstrictors or inotropes. She was extubated after two hours and showed no biochemical evidence of acute kidney injury, with creatinine levels remaining between 0.69 and 1.1 mg/dL. Lactate values remained low, 1.1 ± 0.3 mmol/L, confirming the absence of significant oxygen debt. Fluid balance was favorable, with a minimal postoperative weight increase from 125 to 128 kg. Her respiratory course was uncomplicated, and she was discharged from the intensive care unit after two days with a smooth overall recovery. This clinical evolution supports the effectiveness of a structured, DO₂-centered perfusion strategy in maintaining aerobic metabolism and reducing postoperative complications in an obese patient undergoing complex redo cardiac surgery (Tables 3-4).

**Table 3:** Routine Laboratory Examinations at Key Timepoints

Parameter	Reference Range	Pre-operative	CPB (Pump)	Post-30 min	Post-12 h
Hemoglobin (g/dL)	12-16	13.4	10.4	11.2	9.7
Hematocrit (%)	36-46	40.4	31	32.6	29.1
Platelets (×10³/µL)	150-400	196	130	263	196
Leucocytes (×10³/µL)	4-11	4.77	3.6	21.73	10.31
Urea (mg/dL)	15-45	33.17	28.46	31.89	42.8
Creatinine (mg/dL)	0.6-1.3	0.68	0.59	0.69	1.1
Lactate (mmol/L)	0.5-2.0	1.79	1.1	2.47	1.1
pH	7.35-7.45	7.41	7.45	7.4	7.38
PaO₂ (mmHg)	80-100	79.9	335	200	99.4
PaCO₂ (mmHg)	35-45	37.7	39	42.2	43.2

**Table 4:** Operative and Postoperative Course

Parameter	Value
Cardioplegia	Del Nido
Priming Volume	1150 mL
Hemofiltration	No
Device Deficiencies	None
Heparin Dose	400 U/kg
Protamine Dose	600 U/kg
Urine Output (Intraoperative)	850 mL
Chest Drainage	300 mL + 100 mL
Fluid Balance	-170 mL
Extubation Time	2 hours
Intensive Care Unit Stay	2 days

## Discussion

This case highlights several important considerations in the management of oxygen delivery during CPB in obese patients, a population known to exhibit distinct metabolic and microcirculatory vulnerabilities [1,3]. Obesity is associated with increased whole-body VO₂i, reduced microvascular reserve, impaired endothelial responsiveness, and diminished tolerance to hemodilution [4]. These physiological constraints narrow the safety margin during CPB and increase the risk of entering low indexed oxygen-delivery (DO₂i) states [5].

Traditional perfusion strategies that rely on fixed flow indices, intermittent laboratory measurements, or generic hemodynamic targets may fail to detect early declines in oxygen delivery. This limitation is particularly relevant in patients with elevated BMI, whose metabolic requirements are not accurately captured by conventional flow formulas [6]. In this case, a continuous, integrated, multiparametric DO₂-guided approach proved essential for anticipating metabolic instability and preventing oxygen-debt accumulation [7]. Beyond real-time DO₂i monitoring, the perfusion team tracked additional metabolic parameters, including carbon-dioxide production (VCO₂i), VO₂i, the VO₂/DO₂ ratio, O₂ERi, and the DO₂/VCO₂ perfusion ratio, which collectively provide a sensitive assessment of aerobic reserve and perfusion adequacy.

A central component of this strategy was the incorporation of Area Under the Curve for oxygen delivery (AUC-DO₂), an emerging metric that quantifies both the duration and intensity of exposure to critically low DO₂i levels. Unlike instantaneous measurements, AUC-DO₂ reflects the cumulative biological impact of insufficient oxygen delivery, which better corresponds to the development of cellular oxygen debt and subsequent organ dysfunction [8]. In this patient, adherence to AUC-DO₂ monitoring kept cumulative oxygen-debt exposure below the protective benchmark of 1,000 mL/min/m²/min, with only 8.5 minutes spent below the critical DO₂i threshold of 280 mL/min/m². These findings underscore the importance of minimizing both the depth and duration of low-DO₂ events to prevent conversion to anaerobic metabolism.

Temperature-corrected DO₂ was another critical element of the predictive perfusion approach. At 30°C systemic temperature, metabolic activity decreases proportionally according to Q₁₀ coefficients. The Quantum Perfusion System’s Formula Editor enabled real-time computation of temperature-adjusted DO₂i, displayed alongside standard 37°C-referenced DO₂i values. This dual-parameter visualization prevented misinterpretation of perfusion adequacy and ensured that interventions were based on true metabolic demand rather than misleading absolute values.

The physiology-based perfusion paradigm also improved transfusion decision-making [1,9]. Rather than relying on fixed hemoglobin thresholds, transfusion was guided by maintaining DO₂i above target and preventing AUC-DO₂ accumulation. This individualized approach minimized unnecessary blood administration while sustaining adequate oxygen-carrying capacity, an especially relevant consideration in obese patients who are more susceptible to hemodilution [10].

Stable oxygen delivery throughout CPB translated into favorable postoperative outcomes. Lactate remained low, indicating sustained aerobic metabolism; renal function was preserved without acute kidney injury; and the patient achieved early extubation and hemodynamic stability without vasoconstrictors or inotropes. Minimal postoperative fluid retention and a short ICU stay further demonstrate the advantages of a DO₂-centered perfusion strategy in obese individuals undergoing complex cardiac surgery [3,4].

This case also contributes to the growing body of evidence suggesting that higher DO₂i thresholds, approximately 300 mL/min/m², may be necessary in patients with severe obesity. Existing literature recommends minimum thresholds of 260-280 mL/min/m², but these values are derived largely from heterogeneous or predominantly non-obese cohorts and may underestimate the oxygen requirements of high-BMI patients [1]. Their elevated VO₂i, increased CO₂ kinetics, reduced microvascular responsiveness, and earlier transition to anaerobic metabolism all support the use of higher DO₂-related targets, stricter extraction ratios, and closer metabolic surveillance.

Despite these insights, significant knowledge gaps remain. To date, no dedicated studies have established DO₂i thresholds specific to obese populations, nor have optimal limits been defined for O₂ERi, the DO₂/VCO₂ ratio, the VO₂/DO₂ ratio, or AUC-DO₂ exposure in this subgroup [8,11,12]. Most goal-directed perfusion (GDP) frameworks still treat obesity as a homogeneous extension of the general population, leading clinicians to extrapolate thresholds that may not sufficiently protect patients with severe obesity [1,2]. These limitations highlight the need for prospective research integrating continuous multiparametric DO₂ monitoring, temperature-adjusted modeling of oxygen delivery, and cumulative oxygen-debt analysis.

In summary, this case demonstrates that continuous, multiparametric, temperature-corrected, AUC-integrated DO₂-guided perfusion offers substantial advantages in maintaining metabolic stability during CPB in obese patients. By enabling early detection of deteriorating oxygen delivery, supporting physiology-driven interventions, and preventing cumulative oxygen debt, DO₂-centered strategies represent a meaningful evolution in modern perfusion practice, particularly for high-BMI individuals who are most vulnerable to low-DO₂ events. Further investigation is needed to define evidence-based DO₂ thresholds and develop perfusion protocols tailored to the unique metabolic profile of obese surgical populations.

## Conclusions

This case demonstrates that DO₂i-guided perfusion is a practical and effective strategy for maintaining metabolic stability in obese patients undergoing complex cardiac surgery. Continuous monitoring of oxygen delivery allowed early identification of subcritical DO₂i trends and enabled timely adjustments in pump flow, venous drainage, and hemoglobin support, preventing oxygen debt and sustaining fully aerobic metabolism throughout CPB. The patient’s favorable outcome, characterized by stable lactate levels, preserved renal function without AKI, and an uncomplicated postoperative course, highlights the clinical relevance of a proactive, oxygen-delivery-centered approach in this high-risk population. Given the increased metabolic demand and reduced physiologic reserve associated with obesity, these findings suggest that higher DO₂i targets may be beneficial. However, dedicated research is still lacking, and the optimal DO₂ thresholds for obese patients remain undefined. Further studies are needed to establish evidence-based DO₂i targets and refine perfusion strategies tailored to the specific metabolic profile of high-BMI individuals.
